# Effect of Copolymer Properties on the Phase Behavior of Ibuprofen–PLA/PLGA Mixtures

**DOI:** 10.3390/pharmaceutics15020645

**Published:** 2023-02-14

**Authors:** Anton Iemtsev, Martin Klajmon, Fatima Hassouna, Michal Fulem

**Affiliations:** 1 Department of Physical Chemistry, University of Chemistry and Technology, Prague, Technická 5, 166 28 Prague, Czech Republic; 2 Faculty of Chemical Engineering, University of Chemistry and Technology, Prague, Technická 5, 166 28 Prague, Czech Republic

**Keywords:** biodegradable polymers, PLGA, PLA, amorphous solid dispersion, API–polymer compatibility, PC-SAFT, phase diagrams

## Abstract

Prediction of compatibility of the active pharmaceutical ingredient (API) with the polymeric carrier plays an essential role in designing drug delivery systems and estimating their long-term physical stability. A key element in deducing API–polymer compatibility is knowledge of a complete phase diagram, i.e., the solubility of crystalline API in polymer and mutual miscibility of API and polymer. In this work, the phase behavior of ibuprofen (IBU) with different grades of poly(D,L-lactide-co-glycolide) (PLGA) and polylactide (PLA), varying in composition of PLGA and molecular weight of PLGA and PLA, was investigated experimentally using calorimetry and computationally by the perturbed-chain statistical associating fluid theory (PC-SAFT) equation of state (EOS). The phase diagrams constructed based on a PC-SAFT EOS modeling optimized using the solubility data demonstrated low solubility at typical storage temperature (25 °C) and limited miscibility (i.e., presence of the amorphous–amorphous phase separation region) of IBU with all polymers studied. The ability of PC-SAFT EOS to capture the experimentally observed trends in the phase behavior of IBU–PLA/PLGA systems with respect to copolymer composition and molecular weight was thoroughly investigated and evaluated.

## 1. Introduction 

The poor bioavailability of many active pharmaceutical ingredients (APIs) represents one of the major challenges to successful pharmaceutical development [1]. Several strategies to tackle this problem have been developed, including the formation of polymeric amorphous solid dispersions (ASDs) [2], which consist of amorphous API molecularly dispersed in the polymer matrix, and the preparation of polymeric micro- and nanoparticles [3]. The design of these drug formulations is still governed to a large extent by trial-and-error approaches. An influential parameter determining the successful polymeric drug formulations, their physical stability, and performance-related characteristics is the compatibility of API with the polymer. The compatibility between API and polymer, which at the molecular level reflects the strength of their intermolecular interactions compared to API–API and polymer–polymer cohesive interactions, affects the phase behavior of API–polymer mixtures, such as the size of the immiscibility region (i.e., liquid–liquid equilibria (LLE)) or the solubility of crystalline API in the polymeric matrix (solid–liquid equilibrium (SLE)). SLE determines the maximum concentration of amorphous API that can be thermodynamically stabilized against the recrystallization in the polymer matrix, while LLE identifies the conditions (temperature and concentration) at which liquid–liquid or amorphous phase separation (APS) can occur. Both the recrystallization of API and APS can lead to undesirable heterogeneities of ASD formulations. An initial step in the polymeric drug formulation development is the selection of a suitable polymeric excipient, which must be biocompatible and offer the desired formulation properties such as optimal API loading and physical stability over time. The latter properties are directly related to the compatibility of drug formulation components. As the number of initially identified biocompatible polymers for a given API can be high, it is highly desirable to develop efficient computational tools that would be capable of estimating or ranking the polymer compatibility with a given API. 

Among various polymeric carriers, aliphatic polyesters have received a growing interest in the development of efficient drug delivery systems due to their unique properties including biocompatibility, biodegradability, and low toxicity. Within the biodegradable polyesters, poly(D,L-lactide-co-glycolide) (PLGA) and polylactide (PLA) represent particular relevance in drug delivery [4]. They have been FDA-approved [5]. Their degradation rate by hydrolysis is controlled by regulating the polymer molecular weight, the composition of monomer units in the copolymer (e.g., lactide (LA) to glycolide (GA) ratio in PLGA), and the type of stereo-forms of lactide units (i.e., D- and L-lactide) [6]. PLGA and PLA have proven themselves as effective polymeric carriers in the development of amorphous solid dispersions (ASDs) [7,8] and micro- or nanoparticle-based drug products formulation [9,10,11,12,13]. 

In our previous work [14], in which the phase behavior of various model APIs with two PLGA copolymers possessing a similar molecular weight (about 10,000 g/mol) but different LA to GA ratios (50:50 and 75:25) was studied, it was shown that the perturbed-chain statistical associating fluid theory (PC-SAFT) equation of state (EOS) in connection with calorimetric measurements of SLE curve is capable of predicting a complete phase diagram including amorphous phase separation (APS), which was found to be in agreement with a long-term behavior of the prepared formulations. The systems of ibuprofen (IBU) with both PLGA polymers showed very limited miscibility, which was demonstrated experimentally by APS during calorimetric measurements and long-term physical stability studies. This system is therefore ideal for exploring the capability of modeling tools, such as the perturbed-chain statistical associating fluid theory (PC-SAFT) equation of state (EOS), to capture the effect of copolymer molecular weight and composition on the size of immiscibility region (i.e., LLE), which is in API–polymer systems demonstrated by amorphous phase separation (APS). We note that, in general, LLE predictions using thermodynamic models are fairly challenging. The situation becomes even more complicated in the case of asymmetric mixtures such as API–polymer mixtures. In this work, the effect of composition and molecular weight of a range of PLA/PLGA polymers on the phase behavior (both SLE and LLE) of IBU was thoroughly investigated experimentally by calorimetry and computationally using PC-SAFT EOS.

## 2. Materials and Methods 

### 2.1. Materials 

**Figure 1:**
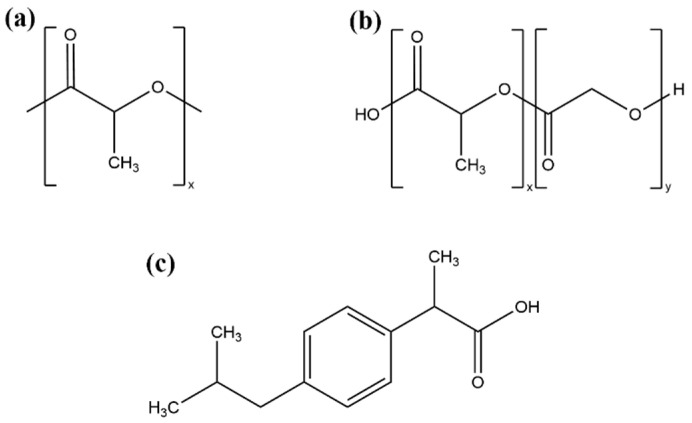
Chemical structures of used compounds: (**a**) PLA, (**b**) PLGA, and (**c**) IBU.

Racemic IBU was kindly donated by Zentiva Group (Prague, Czech Republic). Two PLA commercial grades (Purasorb PDL 02A and PDL 04A) and two PLGA commercial grades (PDLG 5004A and PDLG 7504A) consisting of 50:50 and 75:25 of LA:GA units were graciously provided by Corbion (Gorinchem, The Netherlands). All chemicals were used without further purification. The molecular structures of the used chemicals are depicted in Figure 1, and their selected physicochemical properties are summarized in Section 3.1.

### 2.2. Experimental Methods

#### 2.2.1. Differential Scanning Calorimetry 

The differential scanning calorimetry (DSC) analysis was performed to determine the thermal properties of pure chemical compounds, measure SLE and glass transition temperatures (*T*_g_), and map APS regions. TA Q1000 DSC from TA Instruments, Inc. (New Castle, DE, USA) calorimeter was used. All scans were carried out in the preselected temperature range (−90 to 150 °C) and nitrogen gas purge (50 mL min^−1^). 

The samples (5–10 mg) were placed in aluminum pans, which were tightly sealed and pinned in the lid to permit the evaporation of residual moisture that may be present in the polymer. A thorough calibration of temperature and enthalpy was performed using five reference materials: tin, indium, naphthalene, gallium, and water. 

#### 2.2.2. Calorimetric Determination of SLE Curve and Mapping of LLE Region

The solubility temperature (*T*_s_) of IBU in PLA/PLGA polymers was experimentally determined using DSC. Several DSC protocols are described in the literature and generally applied for the measurement of the *T*_s_: recrystallization (demixing) method [15], melting point depression (MPD) method [16], annealing method [17], melting enthalpy method [18], and step-wise dissolution method (S-WD) [19] (a DSC protocol recently developed in our research group). As the IBU–PLA/PLGA mixtures exhibited APS over a wide range of compositions, the S-WD protocol could not be applied in this work as it derives the solubility from the measured *T*_g_ of a homogenous amorphous system. The MPD method, the most frequently applied DSC protocol, was therefore selected and used in this study. The MPD procedure consists of measuring the melting temperature of API in the polymer matrix, which decreases with the increasing polymer concentration due to the reduction of API chemical potential in the API–polymer mixture. 

The solubility temperatures *T*_s_ of IBU in PLA and PLGA polymers were determined from the melting endotherms of corresponding physical mixtures using the melting peak top (following Höhne et al. [20] recommendation), while the melting temperature (*T*_m_) of the pure IBU was taken as the onset temperature of the melting peak. The physical mixtures were obtained by mild mixing of IBU with PLA/PLGA polymers in predetermined ratios for about 10 min using a pestle and mortar. *T*_s_ of IBU in the PLGA and PLA polymers were measured at the heating rate (*β*) of 1 °C min^−1^ (according to Höhne et al. [20] advising the application of *β* lower than 2 °C min^−1^) during the first heating ramp, which was followed by the cooling (*β* = 20 °C min^−1^) of the melt and second heating ramp (*β* = 10 °C min^−1^) for the *T*_g_ determination. To map the immiscibility regions, i.e., the temperature-composition regions where APS occurs, IBU–PLGA and IBU–PLA mixtures with compositions ranging from 10 to 70 wt.% IBU were subject to successive heating–cooling–heating cycles during which *T*_g_ values of the system were measured. The upper temperature of the initial heating cycle was set slightly above *T*_m_ of IBU to 80 °C. Upper heating temperatures were subsequently increased by 10 °C in each successive cycle up to reaching the final temperature of 150 °C. By this procedure, the temperature-concentration region from 10 to 70 wt.% IBU and from 80 °C to 150 °C was screened for each binary system for APS. If only a single *T*_g_ value was obtained during *T*_g_ determination, positioned between the value for pure amorphous IBU and polymer, the system was considered to consist of a single homogenous amorphous phase. If two *T*_g_ values were detected, the system was considered separated into two amorphous phases—IBU-rich and polymer-rich amorphous phases. 

### 2.3. Modeling Approaches

#### 2.3.1. Modeling of Glass-Transition Temperature

The Kwei equation [21] was used to model *T*_g_ lines for IBU–PLA/PLGA mixtures:

(1) $$T_{g}=\frac{w_{1}T_{g1}+kw_{2}T_{g2}}{w_{1}+kw_{2}}+qw_{1}w_{2}$$

where *T*_g1_ and *T*_g2_ are the glass-transition temperatures of the pure components, *w*_1_ and *w*_2_ are the mass fractions of each component, and *k* and *q* are parameters that can be obtained by fitting the experimental *T*_g_ values for a given binary mixture. 

The Kwei equation (Equation (1)) can also be used with the parameter *k* estimated using the Simha–Boyer rule [22], which requires knowledge of *T*_g_ and density values for both pure components, followed by determination of the parameter *q* by fitting it to experimental binary *T*_g_ data. Since this approach leads to higher deviations from experimental *T*_g_ data [23], a simultaneous fitting of both parameters *k* and *q* to experimental *T*_g_ data was used to model *T*_g_ lines in this work.

#### 2.3.2. SLE and LLE Modeling Using PC-SAFT EOS

Assuming that the crystalline phase consists of only pure IBU, the solubility of IBU in PLA/PLGA polymers $x_{\mathrm{IBU}}^{L}$ (IBU mole fraction in the liquid/amorphous phase) can be calculated using the following rigorous thermodynamic relationship:

(2) $$x_{\mathrm{IBU}}^{L}=\frac{1}{\gamma_{\mathrm{IBU}}^{L}}\exp\left[-\frac{\Delta_{\mathrm{fus}}H}{RT}\left(1-\frac{T}{T_{m}}\right)-\frac{1}{RT}\int_{T_{m}}^{T}\Delta_{\mathrm{fus}}C_{p}dT+\frac{1}{R}\int_{T_{m}}^{T}\frac{\Delta_{\mathrm{fus}}C_{p}}{T}dT\right]$$

where *T* is the absolute temperature; *T*_m_ and ∆_fus_*H* are the melting temperature and fusion enthalpy, respectively, of pure crystalline IBU; ∆_fus_*C_p_* is the difference between the isobaric molar heat capacity of the liquid and crystalline phases of IBU; *R* is the universal gas constant; and $\gamma_{\mathrm{IBU}}^{L}$ is the activity coefficient of IBU in the liquid IBU–polymer phase. As can be seen from Equation (2), SLE calculations require two types of thermodynamic data as the input: (i) thermodynamic fusion data for pure IBU (*T*_m_, ∆_fus_*H*, and ∆_fus_*C_p_*) and (ii) the activity coefficient $\gamma_{\mathrm{IBU}}^{L}$ reflecting the intermolecular interaction between the solute (IBU) and solvent (PLA/PLGA polymers). The fusion thermodynamic properties of pure solute are typically obtained experimentally using calorimetry, while the activity coefficient is calculated using an appropriate thermodynamic model (e.g., EOS). 

For LLE calculations, which determine the miscibility regions in IBU–PLA/PLGA systems, the method of alternating tangents [24] was used in this study. When applying this method, only the composition of one of the coexisting liquid phases is sought, making it an efficient approach for LLE calculations in asymmetric mixtures such as API–polymer mixtures. The obtained binodal points have to satisfy the equilibrium condition for two coexisting phases (L1 and L2) given by the following equations:

(3) $$\begin{array}{l} x_{\mathrm{IBU}}^{L1}\gamma_{\mathrm{IBU}}^{L1}=x_{\mathrm{IBU}}^{L2}\gamma_{\mathrm{IBU}}^{L2}\\ x_{\mathrm{polymer}}^{L1}\gamma_{\mathrm{polymer}}^{L1}=x_{\mathrm{polymer}}^{L2}\gamma_{\mathrm{polymer}}^{L2} \end{array}$$

where $\gamma_{i}^{L1}$ and $\gamma_{i}^{L2}$ are IBU and polymer activity coefficients in phases L1 and L2 and $x_{i}^{L1}$ and $x_{i}^{L2}$ are their mole fractions, respectively.

The activity coefficients needed for both SLE and LLE calculations were obtained using the PC-SAFT EOS [25,26] in its copolymer version [27]. A brief description of the PC-SAFT EOS is provided below.

Within the PC-SAFT framework, the reduced residual Helmholtz energy (*a*^res^) of a system is described as a sum of independent contributions:

(4) $$a^{\mathrm{res}}=a^{\mathrm{hc}}+a^{\mathrm{disp}}+a^{\mathrm{assoc}}$$

where *a*^hc^ is the hard-chain contribution accounting for repulsive forces, *a*^disp^ is the contribution for dispersion attractive forces, and *a*^assoc^ is the contribution accounting for association (hydrogen bonding) interactions. In the PC-SAFT concept, each molecule *i* is represented as a chain of *m* spherical segments of the same diameter *σ_i_*. These two parameters are used to calculate the contribution *a*^hc^. The dispersion contribution *a*^disp^ is evaluated using the energy parameter *ε*_i_/*k* (*k* is the Boltzmann constant) and the association contribution *a*^assoc^ using the energy parameter *ε*_i_^assoc^/*k* and the association volume *κ*_i_^assoc^. Besides the five pure-component parameters mentioned above, the number of association sites *N*_i_^assoc^ (electron donors and acceptors) must be specified based on the molecular structure of a given compound. 

The PC-SAFT parameters for IBU and PLA/PLGA polymers utilized in this study are listed in Table 1. The IBU parametrization was performed in our previous study [28] using the liquid density and vapor pressure data for pure IBU in conjunction with the data on IBU solubility in selected organic solvents. IBU was modeled as a self-associating molecule with four association sites (i.e., 2 hydrogen bond (HB) donors and 2 HB acceptors). The PLGA and PLA were modeled via the copolymer version of PC-SAFT [27] in which polymers are considered as a chain of homopolymer units with their individual sets of PC-SAFT parameters. In this modification, the PC-SAFT EOS is able to account for different arrangements of monomers, composition, and molecular weight of copolymers [27,29]. The parameters for homopolymers PLLA and PDLA were obtained from Cocchi et al. [30] and those for PGA from Prudic et al. [29] (see Table 1). The monomer compositions in the copolymers studied are provided in Table 2. Both PLGA and PLA polymers are assumed to be non-associating in these parametrizations. The bond fractions between the monomer unit segments capturing the copolymer arrangements were taken from Prudic et al. [29].

Activity coefficients $\gamma_{i}^{L}$ can be obtained from the PC-SAFT EOS using the following thermodynamic relationships [25]:

(5) $$\ln\varphi_{i}^{L}=a^{\mathrm{res}}+{\left(\frac{\partial a^{\mathrm{res}}}{\partial x_{i}}\right)}_{T,\rho ,x_{k\neq i}}-\sum_{j}\left[x_{j}{\left(\frac{\partial a^{\mathrm{res}}}{\partial x_{j}}\right)}_{T,\rho ,x_{k\neq j}}\right]+Z-1-\ln Z$$

(6) $$\ln\gamma_{i}^{L}=\ln\varphi_{i}^{L}-\ln\varphi_{0,i}^{L}$$

where *a*^res^ is the reduced residual Helmholtz energy (Equation (4)), $\varphi_{i}^{L}$ is the fugacity coefficient of IBU or polymer in the liquid mixture, $\varphi_{0,i}^{L}$ denotes the fugacity coefficient of the pure liquid, *ρ* is the molar density of the system, and *Z* is the compressibility factor. 

Besides pure-component parameters, binary interaction parameter *k*_ij_ correcting the cross-dispersion energy parameter *ε*_ij_/*k* is introduced within the PC-SAFT framework to achieve closer agreement between experimental results for mixtures and phase behavior predictions [25,26]:

(7) $$\frac{\varepsilon_{\mathrm{ij}}}{k}=\sqrt{\frac{\varepsilon_{i}}{k}\frac{\varepsilon_{j}}{k}}(1-k_{\mathrm{ij}})$$

In this work, two variants of PC-SAFT calculations are presented: (i) pure predictions with *k*_ij_ = 0, i.e., the calculation based solely on pure-component parameters; and (ii) calculations with *k*_ij_ values optimized using experimental SLE data for the one binary system IBU–PDLG 7502A [14]. Based on the performance of pure predictions in terms of identifying phenomena, such as APS, or providing qualitative ordering of SLE data, the suitability of PC-SAFT EOS as a computational screening tool for selecting suitable polymeric carriers for further experimental investigation can be evaluated. In scenario (ii), the ability of the PC-SAFT EOS to predict complete phase diagrams based on optimized *k*_ij_ values using SLE data for IBU with only one polymer from the PLA/PLGA family was studied. In other words, the transferability of modeling phase behavior using PC-SAFT EOS and its ability to describe the trends in phase behavior within the PLA/PLGA family were investigated. 

## 3. Results 

### 3.1. Physicochemical Properties of Pure Components

The physicochemical properties of the pure materials utilized in this study are presented in Table 3. Both melting temperature and fusion enthalpy of IBU agree well with the previously published values summarized by Štejfa et al. [31]. The amorphous IBU was obtained by quenching the melt using *β* = 20 °C min^−1^, and its *T*_g_ was measured during subsequent heating by *β* = 10 °C min^−1^. The obtained *T*_g_ value is in alignment with the results found in the literature, which cover the range from −48 to −45 °C [31]. The recorded *T*_g_ of the PLA/PLGA polymers are in good agreement with the general trend typical for this group of polymers described in the literature: *T*_g_ increases with increasing LA content (e.g., from 35.7 °C for PLGA 50:50 (LA:GA) to 45.3 °C for the PLA 100:0 (LA:GA) according to Pyo Park et al. [32]) and molecular weight of the polymer (e.g., from 42.2 °C for 8000 g/mol PLGA to 52.6 °C 110,000 g/mol PLGA, in accordance with Lee et al. [33]). 

### 3.2. IBU–PLA/PLGA Phase Diagrams

**Figure 2:**
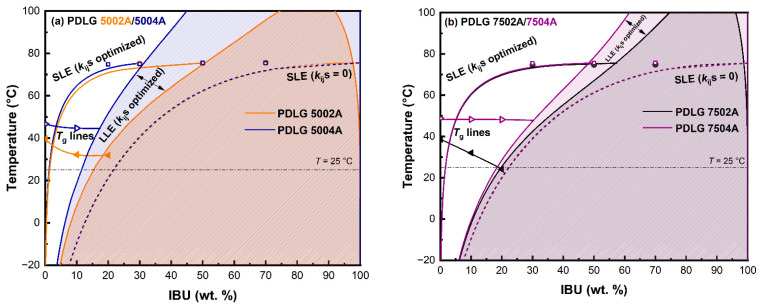

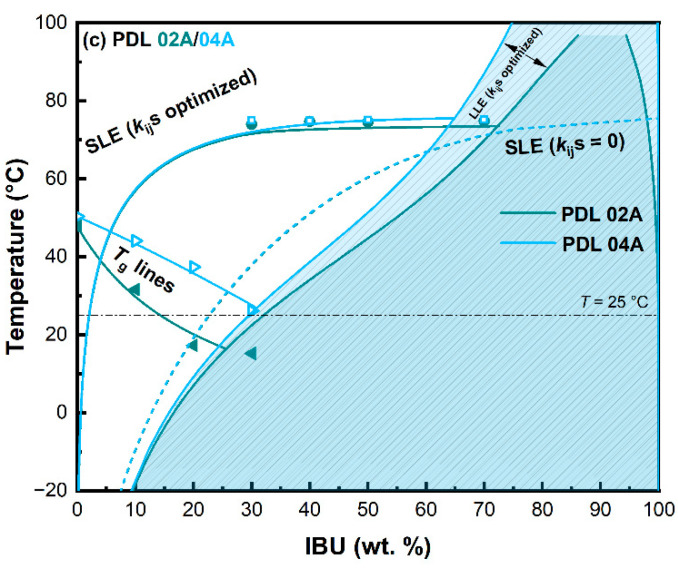
IBU–PLA/PLGA phase diagrams: (**a**) IBU–PDLG 5002A/PDLG 5004A, (**b**) IBU–PDLG 7502A/PDLG 7504A, and (**c**) IBU–PDL 02A/PDL 04A. Symbols represent experimental values and lines represent the calculated properties. *T*_g_ lines were modeled by the Kwei equation, Equation (1), and SLE and LLE using the PC-SAFT EOS. Experimental *T*_g_ and SLE values are listed in Tables S1 and S2, respectively; parameters of the Kwei equation, Equation (1), are listed in Table S3; and applied binary interaction parameters *k*_ij_ are listed in Table S5. Data for IBU–PDLG 5002A and IBU–PDLG 7502A were taken from our previous study [14].

Experimental *T*_s_ and *T*_g_ values for binary systems IBU–PDL 5004A, IBU–PDL 7504A, and IBU–PDL 02A/04A are summarized in Tables S1 and S2 in the Supplementary Material (SM). *T*_s_ and *T*_g_ data for IBU–PDL 5002A and IBU–PDL 7502A were reported in our previous study [14]. The resulting phase diagrams are shown in Figure 2. The *T*_g_ line defining the kinetic stability of ASD formulations was modeled using the Kwei equation (Equation (1)) with parameters listed in Table S3 in SM. The PC-SAFT EOS, which was used to model thermodynamic phase behavior (i.e., SLE and LLE) was applied in two variants: (i) with all binary interaction parameter *k*_ij_s set to zero, which represents pure prediction based solely on pure-component parameters (represented by dashed lines in Figure 2); and (ii) with *k*_ij_ values adjusted based on experimental SLE data for one binary system IBU–PDLG 7502A (represented by solid lines in Figure 2). 

The solubility of IBU in the studied PLA and PLGA polymers obtained by pure predictions at 25 °C (typical storage temperature) is listed in Table 4. As can be seen, with *k*_ij_s = 0, the solubility of IBU was predicted to be almost identical for all polymers examined (regardless of differences in polymer composition and molecular weight) and fell in the range of 21.6 to 22.7 wt.%. The PDL 02A (i.e., lower molecular weight PLA) polymer demonstrated the highest solubility potential for IBU based on pure predictions. Compared to experimental results, the predicted SLE curves considerably overestimated the IBU solubility in all studied polymers. The average absolute relative deviation (AARD) listed in Table S4 ranges from 93 to 157%. Furthermore, and most importantly, PC-SAFT EOS with *k*_ij_s = 0 did not (at least qualitatively) predict the presence of the immiscibility region for any system, which was observed experimentally. 

To achieve an improved description of experimental SLE data using PC-SAFT EOS, the temperature-independent *k*_ij_s between IBU–DLA, IBU–LLA, and IBU–GA (PLGA polymers) and between IBU–LLA and IBU–DLA (PLA polymers) groups, determined based on SLE data for IBU–PDLG 7502A systems in our recent study [14], were applied. In brief, to shorten the number of fitted parameters (following the recommendation of Prudic et al. [29]), *k*_ij_s between the monomeric units of the homopolymer DLA, LLA, and GA were considered to be zero, and *k*_ij_s between IBU–LLA and IBU–DLA were assumed to be identical. The applied *k*_ij_s are listed in Table S5. 

Similar to the pure predictions, the impact of polymer molecular weight and composition on the evaluated solubility was relatively modest (see Table 4). The solubility of IBU at 25 °C predicted using the optimized PC-SAFT EOS was found to range from 1.2 to 2.1 wt.%. As for pure predictions, the solubility of IBU in PDL 02A was predicted to be the highest among the studied polymers. The corresponding AARD values lying in the range from 23.3% (PDL 02A) to 33.4% (PDLG 5004A) are shown in Table S4. Such high AARDs can be explained by the incapacity of PC-SAFT together with *k*_ij_s (temperature-independent) to accommodate a weak dependence of experimental IBU solubility on the temperature in PLA and PLGA polymers (squares and circles in Figure 2). Nonetheless, the PC-SAFT model with the adjusted *k*_ij_s was able to qualitatively predict the presence of liquid–liquid phase splitting events in the IBU–PLA and IBU–PLGA systems, which is in agreement with experimental observations (for details, see Section 3.3).

In accordance with Equation (7), the positive *k*_ij_ denotes the reduction of API–polymer interactions, which, in turn, leads to a decrease in solubility and miscibility. It can be clearly visible for IBU–PLGA and IBU–PLA systems, where relatively high positive values of *k*_ij_ (Table S5) resulted in very limited solubility values (at 25 °C) of the studied systems (see Table 4). Moreover, it can be observed from Figure 2 that, although the polymer composition (i.e., the content of LLA/DLA or GA units) or molecular weight demonstrated only a slight impact on calculated IBU solubility (SLE), the effect on LLE binodal curve was more pronounced, which indicates a higher sensitivity of APS calculations on the model parameters (for details, see Section 3.3). These observations are consistent with the results obtained in the previous study [14] and those by Sadowski and co-authors [29,34]. 

The corresponding *T*_g_ lines were constructed to determine the regions where the IBU–PLGA and IBU–PLA ASD formulations can be stabilized kinetically owing to considerably lower molecular motion at conditions under the *T*_g_ line. As can be seen from the phase diagrams depicted in Figure 2, at a typical storage temperature of 25 °C, ASD formulations with drug loadings higher than a couple of weight percentages are estimated to be thermodynamically unstable as their temperature-composition coordinates are located on the right from the SLE curve. Therefore, the physical stability of ASD formulations (with *w*_IBU_ > 2 wt.% at 25 °C) will be governed by kinetic aspects, i.e., by the difference between the storage temperature and *T*_g_ line. Based on *T*_g_ lines depicted in the phase diagrams (Figure 2), a higher kinetic stabilization of ASD formulations consisting of higher molecular weight polymers can be deduced, which represents an advantage of using higher molecular weight PLA/PLGA polymers in ASD-based drug delivery systems.

### 3.3. Miscibility of IBU with PLA/PLGA Polymers

**Figure 3:**
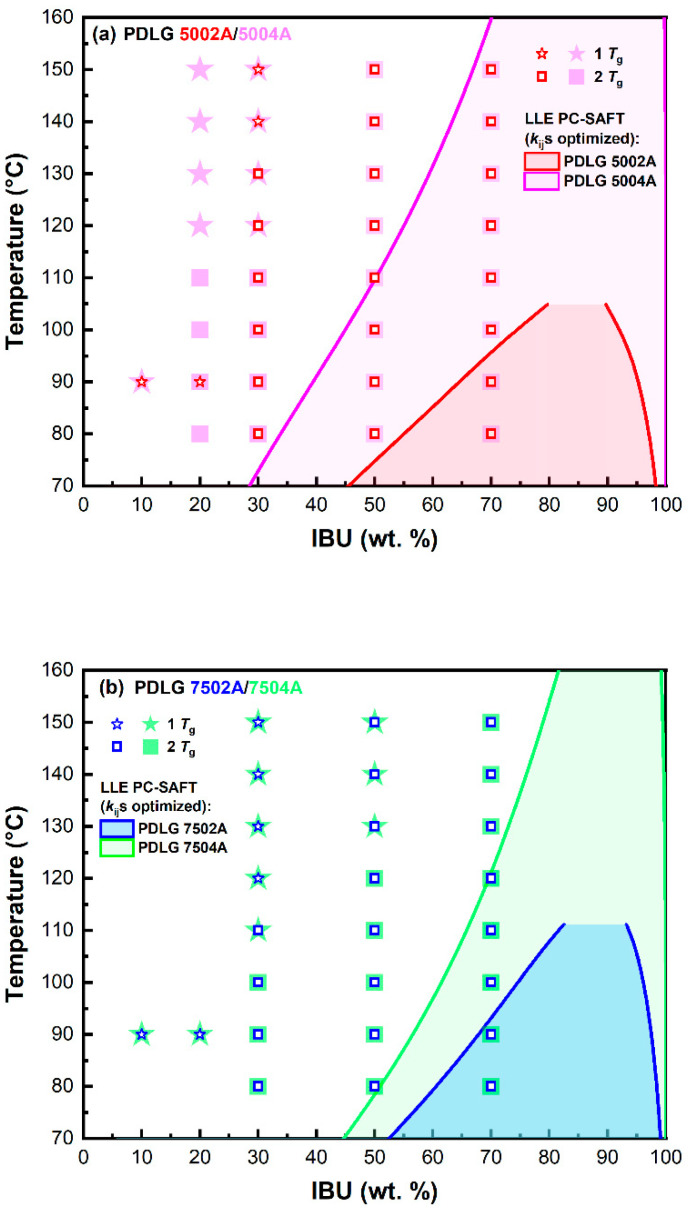

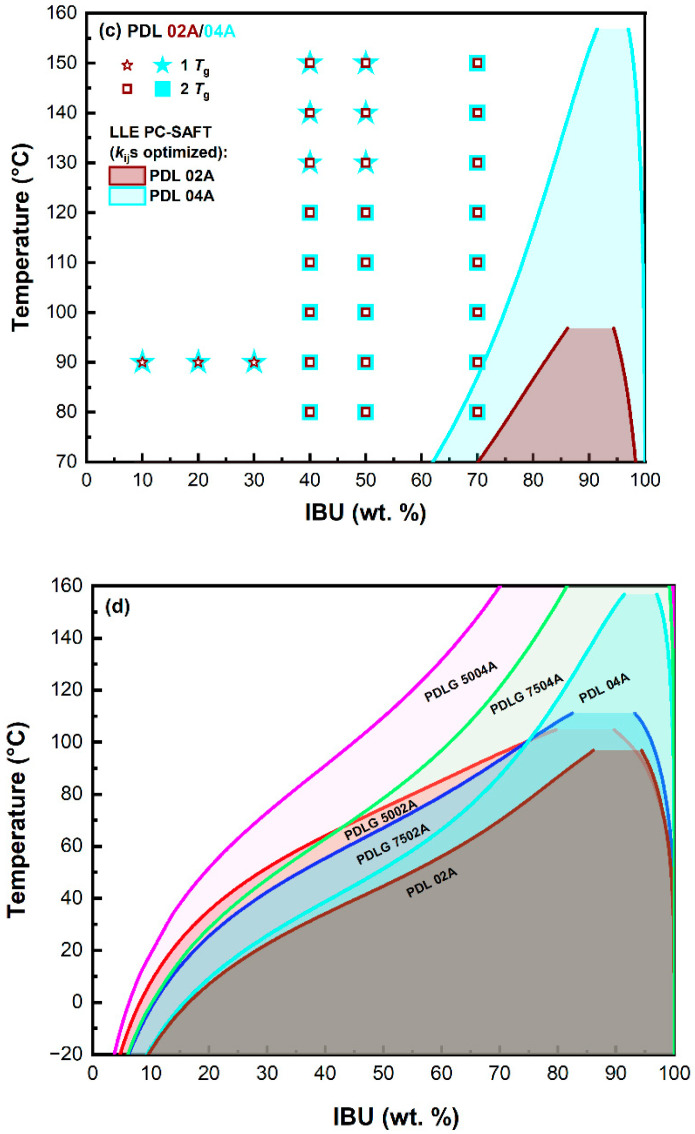
Comparison of LLE areas determined using DSC measurements with those predicted using the PC-SAFT EOS: (**a**) PDLG 5002A/5004A, (**b**) PDLG 7502A/7504A, and (**c**) PDL 02A/04A. (**d**) Comparison of LEE regions modeled by PC-SAFT.

For all binary systems studied, APS (LLE) was observed during the solubility measurements using the MPD method. To outline approximate phase boundaries for APS, i.e., to identify temperature-composition regions at which APS occurs, a series of complementary melt quenching calorimetric experiments was conducted. The calorimetric mapping consisted of the determination of *T*_g_ for binary IBU–PLA/PLGA mixtures, which were annealed at temperatures above IBU melting temperature for fixed composition and subsequently quenched to −90 °C. The maximum annealing temperature was set to 150 °C to avoid the thermal decomposition of IBU. A single *T*_g_ value located between *T*_g_ of pure IBU and pure PLA/PLGA polymer was considered to indicate a single homogenous amorphous mixture, while two *T*_g_ values observed close to *T*_g_ of pure IBU and that of pure polymer were considered to indicate of creation of a two-phase system comprising polymer-rich and IBU-rich phases. The mapping outcomes together with the LLE region predicted using PC-SAFT EOS (optimized *k*_ij_s) are depicted schematically in Figure 3. The *T*_g_ values determined during the mapping experiments are summarized in Table S2. 

Based on the approximate areas of immiscibility regions obtained during the mapping experiments (Figure 3), the following ranking of experimental miscibility of the polymers studied with IBU can be deduced: PDL 04A > PDL 02A > PDLG 7504A > PDLG 7502A > PDLG 5002A > PDLG 5004A. Considering these results, the impact of the polymer composition is clearly visible: the higher LA content, the smaller LLE region (i.e., the higher mutual miscibility of IBU and polymer). At the same time, the impact of the molecular weight of the polymer is not as obvious. In the case of IBU–PDL 04 and IBU–PDLG 7504A systems, the determined LLE region was noticeably smaller compared to the systems of IBU with lower molecular weight analogs (i.e., PDL 02A and PDLG 7502). Hoverer, the situation with PDLG 5002A and PDLG 5004A (i.e., LA to GA ratio 50:50) was the opposite.

The results of LLE modeling using PC-SAFT with optimized *k*_ij_s are presented and compared with experimental results in Figure 3 (modeling with *k*_ij_s = 0 did not indicate LLE). The equilibrium concentrations predicted using the PC-SAFT for the polymer-rich phase at 25 °C are summarized in Table 5. Based on these results, the following miscibility trend was derived: *w*_IBU_ (PDL 02A) > *w*_IBU_ (PDL 04A) > *w*_IBU_ (PDLG 7502A) > *w*_IBU_(PDLG 7504A) > *w*_IBU_ (PDLG 5002A) > *w*_IBU_ (PDLG 5004A). 

For both lower and higher molecular weight polymers, the miscibility between the IBU and polymer increases with increasing LA content, reflecting a higher number of favorable hydrophobic interactions between IBU and LA. These findings qualitatively agree with the experimental results obtained in this work as well as with the results obtained by Luebbert et al. [34] for low molecular weight PLGA and PLA polymers. Regarding the impact of molecular weight at fixed copolymer composition, the experimental mapping of APS suggests that the mutual miscibility of IBU with the polymer increases with increasing polymer molecular weight for polymers with LA content of 75 and 100 mol.%. LLE calculations by PC-SAFT with optimized *k*_ij_s do not capture this trend.

The prediction of the conditions under which the components are miscible is a key parameter in designing homogeneous drug formulations and predicting their long-term physical stability. At the same time, LLE calculations are extremely challenging, especially when considering asymmetrical mixtures such as API–polymer mixtures. As mentioned above, PC-SAFT EOS without optimization using SLE experimental data was unable to signal a possible immiscibility region for the binary systems studied, which is contrary to experimental evidence. This can be considered as a significant limitation if PC-SAFT EOS is to be considered as an in silico screening tool for the selection of suitable polymeric excipients for a given API. In order to detect a possible liquid–liquid split or APS using PC-SAFT EOS, the experimental SLE data should be provided and used for the optimization of binary interaction parameters *k*_ij_ (we note that, in this work, *k*_ij_ values were derived based on SLE data for only one binary system, IBU–PDLG 7502A). With the optimized *k*_ij_ values, PC-SAFT EOS correctly predicted the presence of immiscibility region for all the binary systems studied, although compared to experimental results, the size of heterogonous regions was systematically underestimated. 

## 4. Conclusions

Herein, the thermodynamic phase behavior of IBU with PLA/PLGA polymers of various molecular weights and copolymer compositions was investigated experimentally using calorimetry and computationally using PC-SAFT EOS. Kinetic stability was estimated on the basis of the *T*_g_ line modeled using the Kwei equation. The constructed phase diagrams indicate a very low solubility of IBU in PLA/PLGA polymers at a typical storage temperature of 25 °C, limited miscibility of IBU with PLA/PLGA polymers, and increasing kinetic stability with increasing molecular weight of PLA/PLGA polymers. While the effect of copolymer composition or molecular weight on the solubility of IBU was minimal, it had a more pronounced impact on the size of the miscibility region. The PC-SAFT predictions using solely pure-component parameters for IBU and PLA/PLGA polymers (i.e., pure predictions without any experimental input for a given binary system) significantly overestimated IBU solubility and provided no evidence of possible liquid–liquid split or amorphous phase separation. After optimizing binary interaction parameters *k*_ij_ using SLE experimental data (based on SLE data for one of the six binary systems studied), the PC-SAFT EOS correctly detected a possible formation of a heterogeneous immiscibility region for all the binary systems studied. Using optimized *k*_ij_ values, the PC-SAFT EOS system was able to capture miscibility trends as a function of copolymer composition, while the effect of molecular weight was only partially captured, and the miscibility region was systematically underestimated compared to experimental results. 

## Figures and Tables

**Table 1:** PC-SAFT EOS pure-component parameters.

Compound	${\left(m/M_{w}\right)}_{i}$	$\sigma_{i}$ (Å)	$\varepsilon_{i}$/k (K)	$\varepsilon_{i}^{\mathrm{assoc}}$/k (K)	$\kappa_{i}^{\mathrm{assoc}}$	$N_{i}^{\mathrm{assoc}}$ ^a^
API						
IBU ^b^	0.02636	4.0179	309.40	516.469	0.08946	4 (2, 2)
PLGA ^c^						
PLLA ^d^	0.04545	2.920	230.0	0	0	0 (0, 0)
PDLA ^d^	0.03699	3.120	240.0	0	0	0 (0, 0)
PGA ^e^	0.03130	2.860	233.9	0	0	0 (0, 0)

^a^ $N_{i}^{\mathrm{assoc}}$(D,A) is the total number of hydrogen bonding (HB) sites, D is the number of HB donor sites, and A denotes the number of HB acceptor sites in a molecule. ^b^ Adopted from [28]. ^c^ PLGA and PLA properties are summarized in Table 2 and Table 3. ^d^ Adopted from [30]. ^e^ Adopted from [29].

**Table 2:** Monomer composition (as mass fractions) in the copolymers studied.

Polymer	*w*_LLA_ = *w*_DLA_	*w*_GA_
PDLG 5002A/PDLG 5004A	0.277	0.446
PDLG 7502A/PDLG 7504A	0.394	0.212
PDL 02A/PDL 04A	0.500	0

**Table 3:** Pure-component physiochemical properties.

Compound	*M*_w_^a^ (g·mol^−1^)	*T*_g_^b^ (°C)	*T*_m_^b^ (°C)	Δ*H*_fus_^b^ (kJ·mol^−1^)	Δ*C_p_*_,fus_^c^ (J·K^−1^·mol^−1^)
IBU	206.3	−45.0 ± 0.3	75.6 ± 0.3	26.4 ± 0.8	176.16440 − 0.3449480·(*T*/K)
PDLG 5002A	9877	39.6 ± 0.3	-	-	-
PDLG 5004A	35,900	46.7 ± 0.3	-	-	-
PDLG 7502A	12,900	38.7 ± 0.3	-	-	-
PDLG 7504A	38,800	48.8 ± 0.3	-	-	-
PDL 02A	16,400	47.8 ± 0.3	-	-	-
PDL 04A	40,200	50.4 ± 0.3	-	-	-

^a^ The average molecular weight (*M*_w_) of polymers was determined in this work using gel permeation chromatography (Waters, equipped with a differential refractive index detector) based on narrow polystyrene standards. ^b^ This study except for *T*_g_ values for PDLG 5002A and PDLG 7502A, which were determined in the previous study [14]. Reported uncertainties correspond to the combined expanded uncertainties (*k* = 2, 0.95 level of confidence) in determining temperature and enthalpy values. ^c^ The difference between the isobaric molar heat capacity of a liquid and a crystal. The reported equation was derived based on experimental heat capacities published in [31].

**Table 4:** IBU solubility w_IBU_ (wt.%) in PLA/PLGA polymers at 25 °C calculated using PC-SAFT EOS with k_ij_s = 0 and optimized k_ij_s.

Polymer	*M*_w_ (g·mol^−1^)	LA Group (mol%)	Solubility *w*_IBU_ (wt.%*, k*_ij_s = 0))	Solubility *w*_IBU_ (wt.%, Optimized *k*_ij_s)
Lower *M*_w_ polymers				
PDLG 5002A	9877	50	21.9	1.4
PDLG 7502A	12,900	75	22.4	1.7
PDL 02A	16,400	100	22.9	2.1
Higher *M*_w_ polymers				
PDLG 5004A	35,900	50	21.6	1.2
PDLG 7504A	38,800	75	22.2	1.6
PDL 04A	40,200	100	22.7	2.0

**Table 5:** Miscibility limit w_IBU_ (wt.%, polymer-rich phase) at 25 °C calculated using the PC-SAFT with k_ij_s = 0 and optimized k_ij_s.

Polymer	*M*_w_ (g·mol^−1^)	LA Group (mol%)	APS *w*_IBU_ (wt.%, *k*_ij_s = 0)	APS *w*_IBU_ (wt.%, Optimized *k*_ij_s)
Lower *M*_w_ polymers				
PDLG 5002A	9877	50	-	15.4
PDLG 7502A	12,900	75	-	19.8
PDL 02A	16,400	100	-	31.9
Higher *M*_w_ polymers				
PDLG 5004A	35,900	50	-	11.5
PDLG 7504A	38,800	75	-	18.4
PDL 04A	40,200	100	-	29.6

## Data Availability

All data relevant to the publication are included.

## Supplementary Materials

The following supporting information can be downloaded at: https://www.mdpi.com/article/10.3390/pharmaceutics15020645/s1, Table S1: *T*_s_ values obtained for IBU–PLGA and IBU–PLA systems; Table S2: (a) *T*_g_ values obtained for IBU–PDLG 5004A system annealed at 80–150 °C. (b) *T*_g_ values obtained for IBU–PDLG 7504A system annealed at 80–150 °C. (c) *T*_g_ values obtained for IBU–PDL 02A system annealed at 80–150 °C. (d) *T*_g_ values obtained for IBU–PDL 04A system annealed at 80–150 °C; Table S3: Parameters *k* and *q* of the Kwei equation ((Equation (1) in the main article) for IBU– PLGA and IBU–PLA systems; Table S4: AARD (*w*_API_) values between experimental SLE data and data calculated using PC-SAFT EOS for all IBU–PLGA and IBU–PLA systems; Table S5: PC-SAFT EOS binary interaction parameters, *k*_ij_, between the IBU and the different monomer units of PLA/PLGA polymers.
