# Supplementary material for: Effect of Copolymer Properties on the Phase Behavior of Ibuprofen–PLA/PLGA Mixtures
Source: Pharmaceutics. 2023 Feb 14;15(2):645. doi: 10.3390/pharmaceutics15020645 (PMC9965113; doi:10.3390/pharmaceutics15020645)
Supplement: Supplementary file 1 [file pharmaceutics-15-00645-s001.zip › pharmaceutics-2165496-supplementary.pdf]

pharmaceutics

MDPI

Supplementary Materials

Effect of copolymer properties on the phase behavior of ibuprofen-PLA/PLGA mixtures

Anton Iemtsev[1], Martin Klajmon[1], Fatima Hassouna[2], Michal Fulem[1,*]

[1] Department of Physical Chemistry, University of Chemistry and Technology, Prague, Technická 5, 166 28 Prague 6, Czech Republic
[2] Faculty of Chemical Engineering, University of Chemistry and Technology, Prague, Technická 5, 166 28 Prague 6, Czech Republic
* Correspondence: fulemm@vscht.cz

Table S1. T_s values obtained for IBU–PLGA and IBU–PLA systems.

	**PDLG 5004A**	**PDLG 7504A**	**PDL 02A**	**PDL 04A**
**IBU (wt. %):**		T_s		
20	74.8	–	–	–
30	75.0	75.5	74.0	75.0
40	–	–	74.7	74.9
50	75.5	75.6	74.6	75.1
70	75.4	75.7	74.8	75.2

Table S2(a). T_g values obtained for IBU–PDLG 5004A system anneled at 80-150 °C.

**IBU (wt. %):**	**10**	**20**		**30**		**50**		**70**	
**Temperature (°C):**	T_{g1}	T_{g1}	T_{g2}	T_{g1}	T_{g2}	T_{g1}	T_{g2}	T_{g1}	T_{g2}
80	44.7	−41.4	43.4	−42.3	45.0	−42.7	45.7	−42.5	45.6
90		−41.4	43.0	−41.7	44.4	−42.6	45.8	−42.7	43.2
100		−41.7	42.6	−41.4	43.2	−42.4	36.6	−42.3	41.5
110		−41.2	37.6	−41.6	40.9	−41.9	35.9	−42.4	40.5
120		35.2	–	36	–	−42.1	35.6	−42.1	36.3
130		34.4	–	34.7	–	−42.4	35	−42.3	35.1
140		34.9	–	34.4	–	−42.1	35.3	−42.4	34.9
150		34.3	–	34.9	–	−42.4	35	−41.8	35.3

Table S2(b). T_g values obtained for IBU–PDLG 7504A system anneled at 80-150 °C.

**IBU (wt. %):**	**10**	**20**	**30**		**50**		**70**	
**Temperature (°C):**	T_{g1}	T_{g1}	T_{g1}	T_{g2}	T_{g1}	T_{g2}	T_{g1}	T_{g2}
80	48.3	48.2	−40.7	48.2	−42.1	48.2	−43.2	32.3
90			−41.7	48.4	−41.8	48.6	−43.0	31.5
100			−39.3	46.6	−41.9	47.4	−43.0	31.0
110			40.2	–	−41.3	35.9	−42.8	29.4
120			39.3	–	−41.8	30.0	−42.9	28.5
130			39.7	–	27.5	–	−42.2	27.7
140			40.6	–	27.6	–	−43.4	27.4
150			40.6	–	28.6	–	−44.3	27.8

Table S2(c). T_g values obtained for IBU–PDL 02A system anneled at 80-150 °C.

**IBU (wt. %):**	**10**	**20**	**30**	**40**		**50**		**70**	
**Temperature (°C):**	T_{g1}	T_{g1}	T_{g1}	T_{g1}	T_{g2}	T_{g1}	T_{g2}	T_{g1}	T_{g2}
80	31.6	17.3	15.2	−44.2	12.3	−44.7	13.5	−43.3	12.7
90				−44.7	11.9	−44.3	12.5	−43.2	11.4
100				−44.1	11.0	−44.2	12.5	−43.3	11.5
110				−44.8	12.2	−43.8	13.2	−43.1	12.4
120				−44.7	10.9	−44.1	11.8	−43.1	12.1
130				−44.7	13.0	−44.3	11.1	−43.7	12.4
140				−44.6	13.2	−44.6	11.2	−43.1	11.9
150				−45.6	11.5	−44.6	11.4	−43.4	12.5

Table S2(d). T_g values obtained for IBU–PDL 04A system anneled at 80-150 °C.

**IBU (wt. %):**	**10**	**20**	**30**	**40**		**50**		**70**	
**Temperature (°C):**	T_{g1}	T_{g1}	T_{g1}	T_{g1}	T_{g2}	T_{g1}	T_{g2}	T_{g1}	T_{g2}
80	44.1	37.4	26.1	−44.8	16.4	−43.1	15.4	−42.4	17.1
90				−45.2	14.8	−44.1	15.4	−42.7	15.6
100				−44.9	16.2	−43.8	16.4	−43.6	15.8
110				−45.1	15.7	−44.4	16.6	−44.0	15.2
120				−44.8	14.9	−45.9	18.3	−44.3	14.9
130				16.0	–	16.1	–	−45.1	15.6
140				15.1	–	16.3	–	−44.5	14.5
150				16.1	–	15.8	–	−45.2	16.1

Table S3. Parameters *k* and *q* of the Kwei equation ((Equation (1) in the main article) for IBU–PLGA and IBU–PLA systems.

**Polymer**	***k***	***q***
PDLG 5002A	0.22025	232.97454
PDLG 5004A	0.33251	232.97454
PDLG 7502A	0.99972	14.91052
PDLG 7504A	26.72092	5.39933
PDL 02A	0.24938	117.51721
PDL 04A	0.99909	27.99866

Table S4. AARD (w_{API})[a] values between experimental SLE data and data calculated using PC-SAFT EOS for all IBU–PLGA and IBU–PLA systems.

**Polymer**	**All k_{ij} = 0**	**Optimized k_{ij}s**[b]
PDLG 5002A	109	27.9
PDLG 5004A	157	33.4
PDLG 7502A	93	29.7
PDLG 7504A	121	27.3
PDL 02A	95	23.3
PDL 04A	105	28.6

[a] AARD denotes the average absolute relative deviation: $\mathrm{AARD} = 100N^{-1}\sum_{i=1}^{N}\left|(w_{\mathrm{API}}^{\mathrm{exp}} - w_{\mathrm{API}}^{\mathrm{calc}}) / w_{\mathrm{API}}^{\mathrm{exp}}\right|$; w_{1API} is the API solubility (mass fraction), "exp" and "calc" denote the experimental values and those calculated using PC-SAFT EOS, respectively, and *N* is the number of experimental data points.

[b] The respective optimized k_{ij} values are shown in Table S5.

Table S5. PC-SAFT EOS binary interaction parameters, k_{ij}, between the IBU and the different monomer units of PLGA/PLA polymers.

Polymer	k_{ij} (LLA and DLA)	k_{ij} (GA)
PDLG 5002A/5004A and 7502A/5004A	0.030	0.042
PDL 02A and 04A	0.030	-
